# Endoscopic papillectomy for ampullary tumors: Multicenter retrospective study

**DOI:** 10.1055/a-2863-1302

**Published:** 2026-05-19

**Authors:** Katarzyna Połomska, Nastazja D. Pilonis, Mateusz Szmit, Jakub Krzyzkowiak, Maciej Rupinski, Marcin Romanczyk, Wladyslaw Januszewicz, Andrzej Bialek, Jacek T. Drzewiecki, Piotr Wosiewicz, Lukasz Krupa, Krzysztof Kurek, Tomasz Marek, Michal Kaminski

**Affiliations:** 1 Department of Surgical Oncology, Transplant Surgery and General SurgeryMedical University of GdanskGdanskPoland; 2 Department of Oncological Gastroenterology 49585 The Maria Sklodowska-Curie National Research Institute of Oncology in WarsawWarsawMasovian VoivodeshipPoland; 3 Department of Gastroenterology, Hepatology and Clinical Oncology 37802 Centre for Postgraduate Medical EducationWarszawaPoland; 4 Department of Gastroenterology 382554 Academy of SilesiaKatowiceSilesian VoivodeshipPoland; 5 Department of GastroenterologyPomeranian Medical UniversitySzczecinPoland; 6 Department of Internal Medicine and GastroenterologyProvincial Integrated HospitalElblagPoland; 7 Department of Gastroenterology and Hepatology 49613 Medical University of SilesiaKatowicePoland; 8 Department of Gastroenterology and Hepatology with Internal Disease UnitTeaching Hospital No 1 in RzeszówRzeszowPoland; 9 Medical DepartmentUniversity of RzeszowRzeszowPoland; 10 Department of Gastroenterology and Internal Medicine 37801 Medical University of BialystokBiałystokPoland

**Keywords:** Endoscopy Upper GI Tract, Endoscopic resection (ESD, EMRc, ...), Pancreatobiliary (ERCP/PTCD), ERC topics, GI Pathology

## Abstract

**Background and study aims:**

Although endoscopic papillectomy is an established minimally invasive alternative to surgical resection for ampullary tumors, its technical aspects remain poorly defined. This study aimed to identify factors associated with piecemeal resection and recurrence-free survival after endoscopic papillectomy.

**Patients and methods:**

A multicenter, retrospective analysis of consecutive patients who underwent endoscopic papillectomy in six tertiary Polish centers between 2011 and 2023 was performed. Multivariable logistic and Cox regression models were applied.

**Results:**

A total of 192 patients were included. Adverse events occurred in 26.6% of patients. The most frequent were bleeding (17.3%) and acute pancreatitis (15.6%). En bloc resection was achieved in 66.1% of tumors. Larger tumor size (odds ratio [OR] 1.07 per mm increase; 95% confidence interval [CI] 1.02–1.12]; *P* = 0.004) and submucosal injection (OR 5.50; 95% CI 1.82–16.68; *P* = 0.003) were associated with piecemeal resection. Among 142 patients with a median follow-up of 12 months, recurrence occurred in 40.8%. Tumor size (hazard ratio [HR] 1.04 per mm increase; 95% CI 1.03–1.06; *P* < 0.001) and piecemeal resection (HR 2.37; 95% CI 1.23–4.57; *P* = 0.010) were independently associated with shorter recurrence-free survival.

**Conclusions:**

Larger tumors and piecemeal resection were independently associated with shorter recurrence-free survival. Optimization of technical aspects to achieve higher en bloc resection rates may be critical to reduce recurrence.

## Introduction

Ampullary tumors are increasingly diagnosed due to improvements in accuracy of endoscopic detection technologies 1 . Furthermore, accurate photodocumentation of the major ampulla is one of the quality indicators in upper gastrointestinal endoscopy 2 3 . Hence, ampullary tumors are frequently detected incidentally during upper gastrointestinal endoscopy or as part of routine surveillance in patients with genetic gastrointestinal cancer syndromes, such as familial adenomatous polyposis (FAP). Endoscopic papillectomy has emerged as the primary alternative to surgical resection 4 . Compared with pancreaticoduodenectomy and transduodenal surgical ampullectomy, endoscopic papillectomy offers significant advantages in terms of morbidity, yet risk of adverse events (AEs) remains non-negligible 5 . Technical aspects of endoscopic papillectomy remain poorly defined and are mostly based on expert opinion and small, low-quality reports. These modifiable technical aspects of endoscopic papillectomy may affect both resection completeness and recurrence risk. Current literature consists of small cohort studies and two randomized controlled trials 5 6 7 8 9 10 11 12 13 .

To address these evidence gaps, we conducted a multicenter retrospective analysis of patients who underwent endoscopic papillectomy for ampullary tumors at six tertiary referral centers in Poland, aiming to evaluate technical, patient, and tumor factors associated with piecemeal resection and recurrence-free survival.

## Methods

### Study design

This article was prepared in accordance with the Strengthening the Reporting of Observational Studies in Epidemiology initiative 14 . This was a retrospective, multicenter analysis of patients who underwent endoscopic papillectomy in six tertiary centers in Poland (Warsaw, Katowice, Szczecin, Bialystok, Rzeszow, and Elblag). The Centre of Postgraduate Medical Education acted as the coordinating center for this study, and its Ethics Committee reviewed the protocol and granted a waiver of ethical approval.

### Study population and procedures

We included consecutive patients who underwent endoscopic papillectomy for ampullary tumors between January 1, 2011 and December 31, 2023. All endoscopic procedures were performed by endoscopists with extensive experience in endoscopic retrograde cholangiopancreatography (ERCP) in tertiary referral centers. Use of endoscopic ultrasound (EUS) or cross-sectional imaging before ERCP was at the discretion of the treating physician and local policies. Procedures were performed under conscious sedation or general anesthesia. Endoscopic snare resection of ampullary tumors was performed using a side-viewing therapeutic duodenoscope. Use of submucosal injection, electrocautery settings, or pancreatic stenting was at the discretion of the endoscopist. Management of bleeding and other AEs was at the endoscopist’s and/or center’s discretion. Patients were scheduled for surveillance after endoscopic papillectomy according to local practice (before 2021) or European Society of Gastrointestinal Endoscopy (ESGE) guidelines (from 2021 onward) 4 .

### Histopathology evaluation

After endoscopic biopsy and resection, specimens were collected and processed for histopathological review in accordance with World Health Organization (WHO) guidelines 15 . Histopathological review was completed by expert gastrointestinal pathologists. Dysplasia and cancer were defined according to WHO classification of tumors of the digestive system. Ampullary carcinoma was defined by neoplastic invasion into the submucosa 16 .

### Data extraction

All data were extracted from the centers’ hospital information systems. The collected data included: 1) patient characteristics, including: age, sex, and FAP diagnosis; 2) tumor characteristics: size, intraductal extension, and histopathology; 3) technical aspects of the procedure such as injection, pre-papillectomy cannulation, and pancreatic duct stenting; and 4) AEs: bleeding, perforation, acute pancreatitis, cholangitis, and other AEs. Bleeding was defined as post-procedure bleeding of an intensity requiring management with endoscopic hemostatic techniques. Perforation was defined as a full-thickness defect in the gastrointestinal wall. AEs were graded according to the Adverse events in GastRointEstinal Endoscopy (AGREE) classification 17 .

### Outcomes

The primary long-term outcome of the study was recurrence-free survival. The short-term secondary outcome of the study was piecemeal resection rate. Recurrence was defined as any remnant tissue at the post-resection site with biopsies demonstrating microscopic presence of dysplasia/cancer in any follow-up examination after the procedure. Information on recurrence was obtained from the local hospitals’ databases. The date of the endoscopic procedure was considered the start of the monitored period for recurrence. Diagnosis of recurrence was considered as the event date, and for the remainder of patients, the last date of recorded endoscopic follow-up was considered as the censoring date.

### Statistical analysis

Descriptive statistics were reported using medians and interquartile ranges (IQRs). Baseline characteristics between FAP and sporadic lesions were compared using Mann-Whitney U test. Associations between variables were tested using, chi-squared tests, multivariable logistic regression, and Cox proportional hazards regression models. All hypotheses were tested at the 5% significance level.

Factors associated with the secondary outcome were analyzed using multivariable logistic regression. We applied stepwise selection of variables with a P value threshold of 0.05 for entry and 0.10 for removal. To account for potential inter-center variability, a mixed-effects logistic regression model was fitted with study center included as a random effect.

Factors associated with recurrence-free survival were analyzed using Cox proportional hazards regression. To account for potential inter-center variability, robust standard errors were clustered by study center. For multivariable Cox proportional hazards analyses, we applied stepwise selection of variables with a P value threshold of 0.05 for entry and 0.10 for removal.

Candidate predictor variables were selected a priori based on clinical reasoning and previously reported associations. A conceptual framework illustrating hypothesized relationships was created ( **Supplementary Fig. 1** ). Patient-related variables included age, sex, and FAP status. Tumor-related variables included tumor size, tumor histologic grade, and intraductal extension. Procedure-related variables included submucosal injection, pre-papillectomy cannulation, en bloc versus piecemeal resection, and previous resection attempts.

Analyses were performed using Stata 18 (StataCorp LLC, College Station, Texas, United States) and Python 3.11.3 in the JupyterLab 3.6 environment.

## Results

### Baseline characteristics

In total, 192 patients with ampullary tumors were included in this study. Median age of patients was 62 years (interquartile range [IQR] 49–71) and 41.7% were male. Jaundice before endoscopic papillectomy was reported in 18 patients (10%). Median maximal diameter of tumors was 12 mm (IQR 8–20 mm). Pretreatment EUS was performed in 57 patients (29.7%). Pretreatment biopsies were performed in 172 patients (89.5%). Prior endoscopic resection attempts had been performed in 22 patients (11.5%). In preprocedural staging, 58 patients (30.2%) underwent abdominal computed tomography (CT)/magnetic resonance imaging (MRI), and 57 (29.7%) underwent EUS.

There were 39 (20%) FAP-related tumors and 153 (80%) sporadic tumors. Tumors in patients with FAP were smaller (7 vs. 15 mm, P < 0.01), did not have intraductal extension, and 92% of these tumors had low-grade dysplasia (LGD) on pretreatment biopsy. All baseline characteristics of the included patients are presented in Table 1 .

**Table:** **Table 1** Baseline characteristics.

Variable		Patients, n (%)	Total	Missing
Age		62.0 (49.0–71.0)	192	0
Sex	F	112 (58.3%)	192	0
	M	80 (41.7%)		
Jaundice	No	162 (90.0%)	180	12
	Yes	18 (10.0%)		
FAP	No	153 (79.7%)	192	0
	Yes	39 (20.3%)		
Previous attempt	No	170 (88.5%)	192	0
	Yes	22 (11.5%)		
Pretreatment EUS	No	135 (70.3%)	192	0
	Yes	57 (29.7%)		
Pretreatment biopsy results	Benign	22 (12.8%)	172	20
	Adenocarcinoma	3 (1.7%)		
	HGD	29 (16.9%)		
	LGD	117 (68.0%)		
	NEN	1 (0.6%)		
Intraductal extension	No	83 (82.2%)	101	91
	Yes	18 (17.8%)		
Abdominal CT/MR	No	134 (69.8%)	192	0
	Yes	58 (30.2%)		
Tumor size		12.0 (8.0–20.0)	183	9
Pre-papillectomy cannulation	No	115 (59.9%)	192	0
	Yes	77 (40.1%)		
Injection	No	166 (86.5%)	192	0
	Yes	26 (13.5%)		
Attempted pancreatic duct stenting	No	31 (16.1%)	192	0
	Yes	161 (83.9%)		
Pancreatic duct stenting success	No	32 (19.9%)	161	31
	Yes	129 (80.1%)		
Rectal diclofenac	No	39 (21.9%)	178	14
	Yes	139 (78.1%)		
Center	1	3 (1.6%)	192	0
	2	8 (4.2%)		
	3	36 (18.8%)		
	4	27 (14.1%)		
	5	40 (20.8%)		
	6	78 (40.6%)		
CT, computed tomography; HGD, high-grade dysplasia; LGD, low-grade dysplasia; MR, magnetic resonance; NEN, neuroendocrine neoplasm.				

### Procedure characteristics

Pre-papillectomy cannulation was performed on 77 patients (40.1%). Pancreatic duct stenting was attempted in 161 patients (83.9%), of whom success was achieved in 129 (80.1%). Submucosal injection was performed in 26 patients (13.5%). Rectal diclofenac was administered to 139 of 178 patients with available data (78.1%).

### Adverse events

AEs were reported in 51 patients (26.6%) in this cohort ( Table 2 ). Bleeding occurred in 33 patients (17.3%), acute pancreatitis occurred in 30 patients (15.6%), and cholangitis occurred in five patients (2.6%). Perforation occurred in two patients (1%).

**Table:** **Table 2** Procedure outcomes, adverse events, histopathology, and follow-up data.

Variable		Patients, n (%)	Total	Missing
Bleeding	No	158 (82.7%)	191	1
	Yes	33 (17.3%)		
Perforation	No	190 (99.0%)	192	0
	Yes	2 (1.0%)		
Resection	Piecemeal	65 (33.9%)	192	0
	En bloc	127 (66.1%)		
Acute pancreatitis	No	162 (84.4%)	192	0
	Yes	30 (15.6%)		
Cholangitis	No	187 (97.4%)	192	0
	Yes	5 (2.6%)		
Adverse events according to AGREE classification	0	141 (73.4%)	192	0
	1	0 (0%)		
	2	20 (10.4%)		
	3a	18 (9.4%)		
	3b	3 (1.6%)		
	4a	9 (4.7%)		
	4b	1 (0.5%)		
	5	0 (0%)		
Histopathology of resected tumor	Benign histology	32 (17.9%)	179	13
	Adenocarcinoma	10 (5.6%)		
	HGD	26 (14.5%)		
	LGD	110 (61.5%)		
	NEN	1 (0.6%)		
Accuracy of initial staging	Accurate	120 (75.5%)	159	33
	Downstaging	21 (13.2%)		
	Upstaging	18 (11.3%)		
Endoscopic follow-up	No	50 (26.0%)	192	0
	Yes	142 (74.0%)		
Follow-up duration		12.0 (4.9–30.7)	142	50
Recurrence	No	84 (59.2%)	142	50
	Yes	58 (40.8%)		
Treatment of recurrence	Argon plasma coagulation	3 (5.2%)	58	134
	Endoscopic mucosal resection	23 (39.7%)		
	Radiofrequency ablation	4 (6.9%)		
	Surgery	17 (29.3%)		
	None	11 (19.0%)		
AGREE, Adverse events in GastRointEstinal Endoscopy; HGD, high-grade dysplasia; LGD, low-grade dysplasia; NEN, neuroendocrine neoplasm.				

### Tumor histology

Histopathological examination results were available for 179 (93%) resected specimens. In 13 cases (6.8%), histopathological examination was not feasible due to thermal damage to the specimen. Of the examined specimens, invasive adenocarcinoma was diagnosed in 10 cases (5.6%), high-grade dysplasia (HGD) in 26 cases (14.5%), LGD in 110 cases (61.5%), neuroendocrine neoplasm (NEN) in one case (0.6%), and benign histology in 32 cases (17.9%) ( Table 2 ). None of the patients with FAP had invasive adenocarcinoma. Pretreatment histopathological evaluation of specimens revealed invasive adenocarcinoma in three cases (1.7%), NEN in one case (0.6%), HGD in 29 cases (16.8%), LGD in 117 cases (68.0%), and benign histology in 22 cases (12.8%). Of the 159 patients who had results of both pretreatment biopsy and papillectomy histopathological examinations, 120 (75.5%) were initially accurately staged, 21(13.2%) were initially down-staged, and 18 (11.3%) were initially up-staged.

### Short-term outcome

#### En bloc resection

**Fig. 1:**
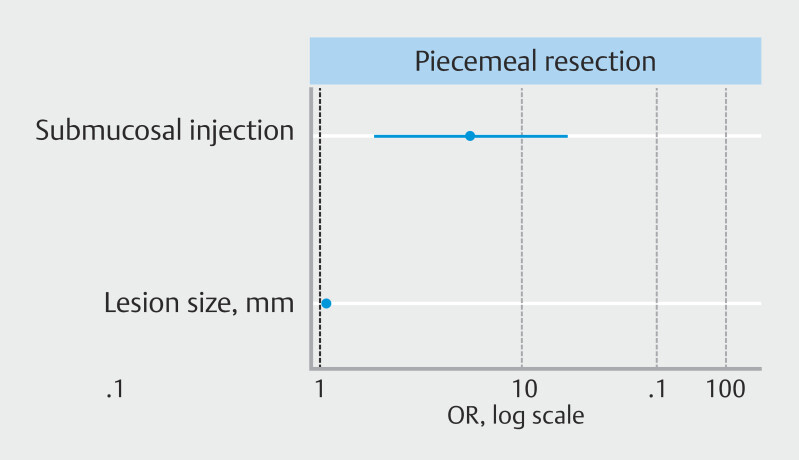
Forest plots of multivariable logistic regression model for piecemeal resection.

En bloc resection was achieved in 127 cases (66.1%) and the remaining 65 patients (33.9%) had piecemeal resection ( Table 2 ). The following variables were considered as potentially associated with piecemeal resection: submucosal injection, tumor size, previous treatment attempt, pre-papillectomy cannulation, age, sex, and FAP. Intraductal extension was excluded because of substantial missing data. In stepwise logistic regression analysis, submucosal injection and tumor size were retained. In the mixed-effects logistic regression model with center included as a random effect, submucosal injection (odds ratio [OR] 5.50; 95% confidence interval [CI] 1.82–16.68; P = 0.003) and tumor size (OR 1.07 per mm increase; 95% CI 1.02–1.12; P = 0.004) were independently associated with piecemeal resection ( Fig. 1 ), with no significant inter-center variability.

### Long-term outcome

#### Recurrence

**Fig. 2:**
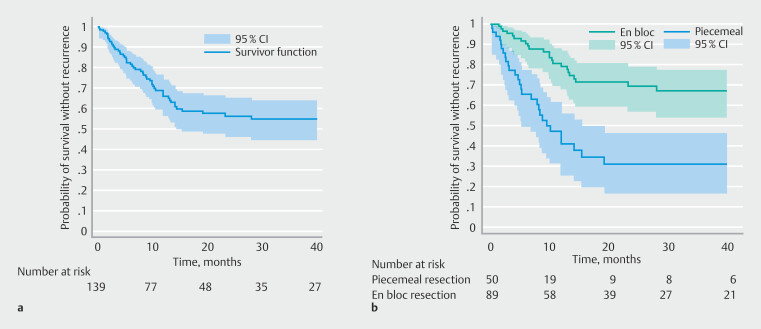
Kaplan-Meier estimates of recurrence-free survival after endoscopic papillectomy. **a** Overall recurrence-free survival for the entire cohort. **b** Recurrence-free survival stratified by type of resection (en bloc vs. piecemeal).

Endoscopic follow-up data were available for 142 patients (74.0%). Median follow-up duration was 12.0 months (IQR 4.9–30.7). Recurrence was observed in 58 patients (40.8%) with endoscopic follow-up ( Table 2 ). Median time to recurrence was 8.3 months (IQR 3.1–13.1). Kaplan-Meier analysis demonstrated a recurrence-free survival rate of 66.0% (95% CI 56.6%-73.8%) at 12 months and 56.3% (95% CI 46.3%-65.1%) at 24 months ( Fig. 2 **a** ). The following variables were considered as potentially associated with recurrence: previous treatment attempt, piecemeal resection, tumor histologic grade, tumor size, age, sex, and FAP. Intraductal extension was excluded because of substantial missing data. In the stepwise Cox regression analysis, piecemeal resection and tumor size were retained. In the Cox regression analysis with robust standard errors clustered by study center, piecemeal resection (hazard ratio [HR] 2.37; 95% CI 1.23–4.57; P = 0.010), and tumor size (HR 1.04 per mm increase; 95% CI 1.03–1.06; P < 0.001) were independently associated with recurrence risk. Proportional hazards assumption, tested using Schoenfeld residuals, was not violated. Kaplan-Meier curves stratified by en bloc vs. piecemeal resection are presented in Fig. 2 **b** .

### Inter-center variability

We observed inter-center variability in percentage of patients with FAP (P < 0.001) and tumor size (P < 0.001). This could have influenced differences in the outcomes. Centers with a higher proportion of FAP patients with smaller tumors less frequently had invasive adenocarcinoma diagnosed, less frequently observed post-procedure bleeding, and followed-up a higher proportion of patients. In terms of technical differences in endoscopic papillectomy, inter-center variability was observed in pre-papillectomy cannulation (P = 0.002), injection rates (P < 0.001), pancreatic duct stenting success (P = 0.014), and administration of rectal diclofenac (P < 0.001). Preprocedural staging (abdominal CT/MR and EUS) significantly differed between centers (P < 0.001). Inter-center variability was further observed in the tumor histological grade (P < 0.001) and accuracy of initial staging (P < 0.001). Recurrence rates varied significantly (P = 0.019), probably due to differences in the follow-up duration (P = 0.01), Detailed data are available in **Supplementary Table 1** .

## Discussion

This multicenter retrospective study provides a comprehensive assessment of real-world practices and technical aspects of endoscopic papillectomy for ampullary tumors across Polish tertiary endoscopic centers. The findings demonstrate both the potential and limitations of endoscopic papillectomy, particularly in the context of inadequate patient selection and inconsistent procedure standards prior to introduction of formal ESGE guidelines in 2021 4 .

Recurrence occurred in over 40% of patients in this cohort. We found that larger tumor size and piecemeal resection were independently associated with shorter recurrence‑free survival after endoscopic papillectomy for ampullary tumors. Submucosal injection was strongly associated with piecemeal resection, which is consistent with current ESGE guidelines discouraging routine submucosal injection 4 . This modifiable technical factor may indirectly affect long‑term outcomes, which confirms previous reports 8 18 .

Frequency of AEs was similar to previously reported rates 19 . In our cohort, endoscopic papillectomy was frequently associated with AEs (26.6%), including pancreatitis (15.6%) and post-procedure bleeding (17.3%). These findings suggest intrinsic morbidity of the procedure itself. Therefore, in cases of tumors without biopsy-proven adenoma, the risk-benefit ratio of endoscopic papillectomy should be carefully weighed. At the same time, limited accuracy of pretreatment biopsies supports ESGE recommendations to complement biopsy with EUS and magnetic resonance cholangiopancreatography for staging and treatment planning 4 .

Furthermore, none of the patients with FAP were diagnosed with invasive adenocarcinoma and the majority (92%) had LGD. These observations support the notion that FAP patients with suspected ampullary tumors without confirmed dysplasia may benefit from active surveillance only 20 . Preprocedure assessment is crucial to identify patients who may benefit the most from endoscopic papillectomy.

Our analysis shows substantial inter-center variability in pre-procedure staging, procedure technical details, and surveillance protocols. This heterogeneity reflects absence of standardized protocols prior to the 2021 ESGE guideline publication 4 .

This study had several limitations. First, follow-up data were incomplete for over one-quarter of the cohort, which may have led to underestimation of recurrence rates.

Furthermore, no formal power calculation was performed a priori. Consequently, estimates have limited precision, as reflected in the wide CIs. These broad CIs should be considered when interpreting the magnitude of associations reported.

However, considering the low incidence of ampullary tumors, and previously reported cohorts 5 6 7 8 9 12 13 19 , our analysis of 192 patients from six tertiary centers provides substantial insight into technical aspects and outcomes of the procedure.

## Conclusions

In conclusion, endoscopic papillectomy is a minimally invasive therapeutic option for ampullary tumors, but is associated with a significant risk of AEs and recurrence. Risk of recurrence can be mitigated with increased en bloc resection rates. Standardized protocols, including adequate qualification and resection methods, are essential for improving outcomes of endoscopic papillectomy.

## Supplementary Material

Supplementary Material Zusatzmaterial
