# Supplementary material for: Endoscopic papillectomy for ampullary tumors: Multicenter retrospective study
Source: Endosc Int Open. 2026 May 19;14:a28631302. doi: 10.1055/a-2863-1302 (PMC13289971; doi:10.1055/a-2863-1302)

Supplementary Table 1 Inter-center variability.

		Center 1	Center 2	Center 3	Center 4	Center 5	Center 6	*P* value
		**N = 3**	**N = 8**	**N = 36**	**N = 27**	**N = 40**	**N = 78**	
FAP	0	2 (67%)	7 (88%)	32 (89%)	27 (100%)	37 (92%)	48 (62%)	**< 0.001**
	1	1 (33%)	1 (12%)	4 (11%)	0 (0%)	3 (8%)	**30 (38%)**	
Lesion size		14.0 (12.0-14.0)	20.0 (20.0-27.5)	10.0 (7.0-12.0)	20.0 (12.0-25.0)	19.0 (14.0-30.0)	**9.0 (7.0-15.0)**	**< 0.001**
EUS	0	1 (33%)	7 (88%)	32 (89%)	13 (48%)	30 (75%)	52 (67%)	**0.006**
	1	2 (67%)	1 (12%)	4 (11%)	**14 (52%)**	10 (25%)	26 (33%)	
CT/MR	0	0 (0%)	2 (25%)	28 (78%)	7 (26%)	27 (68%)	70 (90%)	**< 0.001**
	1	3 (100%)	6 (75%)	8 (22%)	**20 (74%)**	13 (32%)	8 (10%)	
Pre-papillectomy cannulation	0	0 (0%)	5 (62%)	22 (61%)	16 (59%)	15 (38%)	57 (73%)	**0.002**
	1	3 (100%)	3 (38%)	14 (39%)	11 (41%)	**25 (62%)**	21 (27%)	
Injection	0	3 (100%)	6 (75%)	34 (94%)	25 (93%)	24 (60%)	74 (95%)	**< 0.001**
	1	0 (0%)	2 (25%)	2 (6%)	2 (7%)	**16 (40%)**	4 (5%)	
Attempted pancreatic duct stenting	0	0 (0%)	1 (12%)	11 (31%)	3 (11%)	9 (22%)	7 (9%)	0.055
	1	3 (100%)	7 (88%)	25 (69%)	24 (89%)	31 (78%)	71 (91%)	
Pancreatic duct stenting success	0	1 (33%)	2 (25%)	5 (14%)	10 (37%)	8 (20%)	15 (19%)	**0.014**
	1	2 (67%)	5 (62%)	20 (56%)	16 (59%)	27 (68%)	**59 (76%)**	
	Missing	0 (0%)	1 (12%)	11 (31%)	1 (4%)	5 (12%)	4 (5%)	
Rectal diclofenac	0	0 (0%)	0 (0%)	13 (36%)	0 (0%)	6 (15%)	20 (26%)	**< 0.001**
	1	3 (100%)	8 (100%)	23 (64%)	**27 (100%)**	33 (82%)	45 (58%)	
	Missing	0 (0%)	0 (0%)	0 (0%)	0 (0%)	1 (2%)	13 (17%)	
Bleeding	0	3 (100%)	4 (50%)	35 (97%)	17 (63%)	32 (80%)	67 (86%)	**< 0.001**
	1	0 (0%)	3 (38%)	1 (3%)	**10 (37%)**	8 (20%)	11 (14%)	
	NA	0 (0%)	1 (12%)	0 (0%)	0 (0%)	0 (0%)	0 (0%)	
Perforation	0	3 (100%)	8 (100%)	36 (100%)	27 (100%)	40 (100%)	76 (97%)	0.71
	1	0 (0%)	0 (0%)	0 (0%)	0 (0%)	0 (0%)	2 (3%)	
Resection	Piecemeal	0 (0%)	3 (38%)	16 (44%)	10 (37%)	17 (42%)	19 (24%)	0.16
	En bloc	3 (100%)	5 (62%)	20 (56%)	17 (63%)	23 (57%)	59 (76%)	
Final dysplasia	None	0 (0%)	0 (0%)	**17 (47%)**	3 (11%)	4 (10%)	8 (10%)	**< 0.001**
	Adenocarcinoma	0 (0%)	1 (12%)	2 (6%)	1 (4%)	4 (10%)	2 (3%)	
	HGD	0 (0%)	3 (38%)	0 (0%)	3 (11%)	7 (18%)	13 (17%)	
	LGD	3 (100%)	4 (50%)	16 (44%)	19 (70%)	23 (57%)	45 (58%)	
	NA	0 (0%)	0 (0%)	1 (3%)	1 (4%)	1 (2%)	10 (13%)	
	NEN	0 (0%)	0 (0%)	0 (0%)	0 (0%)	1 (2%)	0 (0%)	

Initial upstaging/downstaging	Accurate	2 (67%)	4 (50%)	31 (86%)	21 (78%)	16 (40%)	46 (59%)	**< 0.001**
	Missing	0 (0%)	2 (25%)	2 (6%)	3 (11%)	14 (35%)	12 (15%)	
	Downstaging	1 (33%)	1 (12%)	0 (0%)	2 (7%)	10 (25%)	7 (9%)	
	Upstaging	0 (0%)	1 (12%)	3 (8%)	1 (4%)	0 (0%)	13 (17%)	
Acute pancreatitis	0	2 (67%)	5 (62%)	29 (81%)	23 (85%)	38 (95%)	65 (83%)	0.18
	1	1 (33%)	3 (38%)	7 (19%)	4 (15%)	2 (5%)	13 (17%)	
Cholangitis	0	3 (100%)	7 (88%)	35 (97%)	26 (96%)	39 (98%)	77 (99%)	0.57
	1	0 (0%)	1 (12%)	1 (3%)	1 (4%)	1 (2%)	1 (1%)	
AGREE	2	0 (0%)	1 (12%)	5 (14%)	3 (11%)	1 (2%)	10 (13%)	0.54
	3a	0 (0%)	1 (12%)	0 (0%)	2 (7%)	4 (10%)	11 (14%)	
	3b	0 (0%)	1 (12%)	0 (0%)	0 (0%)	0 (0%)	2 (3%)	
	4a	0 (0%)	1 (12%)	2 (6%)	1 (4%)	1 (2%)	4 (5%)	
	4b	0 (0%)	0 (0%)	0 (0%)	0 (0%)	0 (0%)	1 (1%)	
	Missing	3 (100%)	4 (50%)	29 (81%)	21 (78%)	34 (85%)	50 (64%)	
Endoscopcic follow-up	0	1 (33%)	3 (38%)	19 (53%)	4 (15%)	10 (25%)	24 (31%)	0.062
	1	2 (67%)	5 (62%)	17 (47%)	22 (81%)	30 (75%)	54 (69%)	
	Missing	0 (0%)	0 (0%)	0 (0%)	1 (4%)	0 (0%)	0 (0%)	
Follow-up duration		10.8 (7.4-14.1)	4.8 (4.1-6.4)	10.8 (3.0-23.1)	5.9 (2.8-12.0)	8.0 (3.6-28.6)	20.4 (9.5-56.0)	**< 0.001**
Recurrence	0	1 (33%)	5 (62%)	13 (36%)	13 (48%)	18 (45%)	34 (44%)	**0.019**
	1	1 (33%)	2 (25%)	4 (11%)	11 (41%)	12 (30%)	28 (36%)	
	Missing	1 (33%)	1 (12%)	19 (53%)	3 (11%)	10 (25%)	16 (21%)	

CT, computed tomography; EUS, endoscopic ultrasound; FAP, familial adenomatous papillosis; HGD, high-grade dysplasia; LGD, low-grade dysplasia; MR, magnetic resonance; NA, not applicable; NEN, neuroendocrine neoplasm

Supplemental figure 1

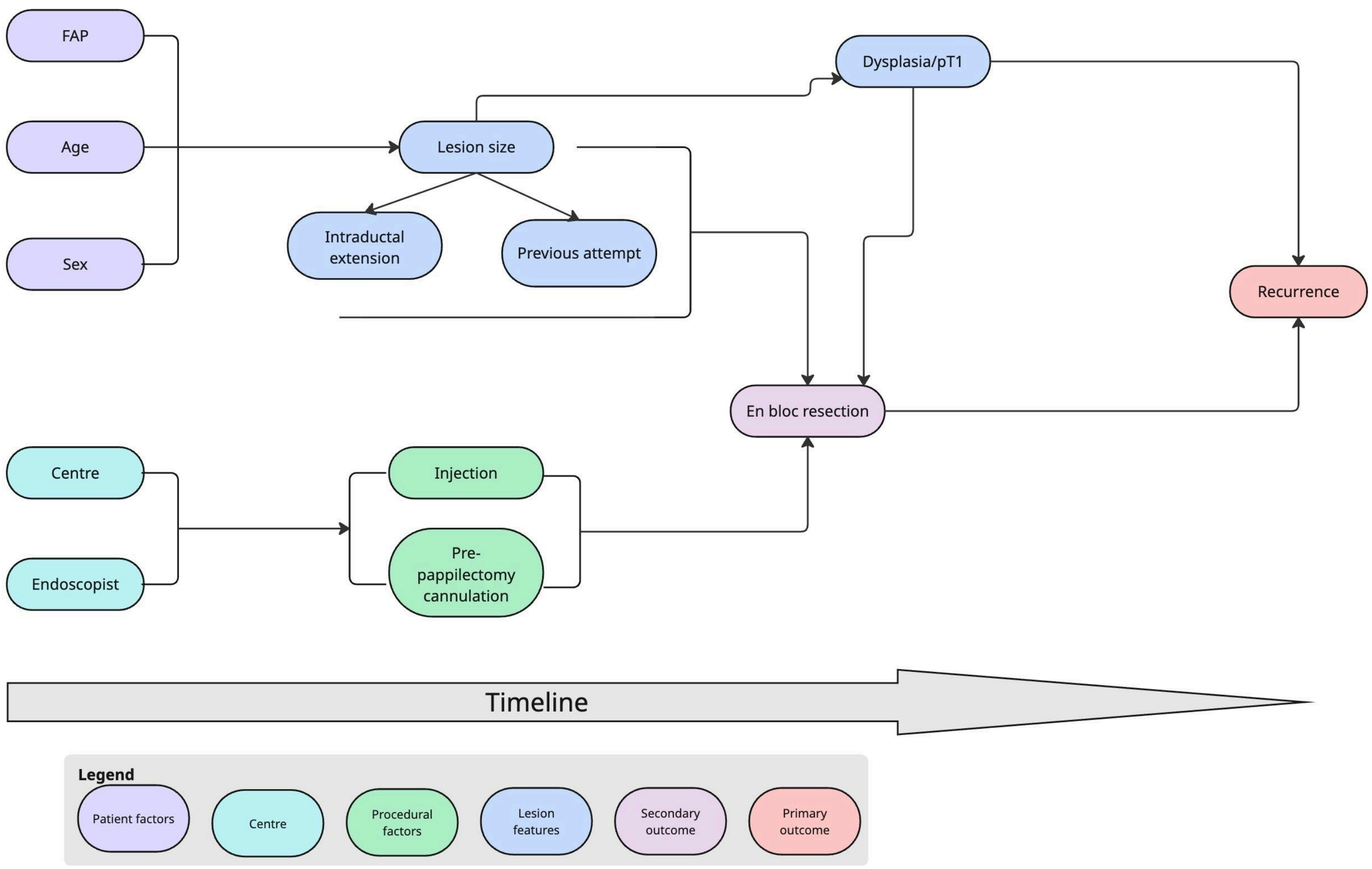

Supplement: Supplementary file 1 — Supplementary Material [file 10-1055-a-2863-1302_28729320.pdf]
